# Anti-Viral Evaluation of Sesquiterpene Coumarins from *Ferula assa-foetida *against HSV-1

**Published:** 2014

**Authors:** Alireza Ghannadi, Khadijeh Fattahian, Yalda Shokoohinia, Mandana Behbahani, Alireza Shahnoush

**Affiliations:** a *Isfahan Pharmaceutical Sciences Research Center, School of Pharmacy and Pharmaceutical Sciences, Isfahan University of Medical Sciences, Isfahan, Iran. *; b *Novel Drug Delivery Research Center *&* Department of Pharmacognosy and Biotechnology, School of Pharmacy, Kermanshah University of Medical Sciences, Kermanshah, Iran. *; c *Department of Biotechnology, Faculty of Advanced Sciences and Technologies, University of Isfahan, Isfahan, Iran. *; d *Department of Chemistry, University of Isfahan, Isfahan, Iran.*

**Keywords:** Kellerin, Badrakemin acetate, Samarcandin diastereomer, Ferula assa-foetida, Herpes simplex

## Abstract

Several complications attributed with Herpes virus related infections and the emergence of drug resistant viruses prompt scientists to search for new drugs. Several terpenoids and coumarins have shown anti HSV effects while no sesquiterpene coumarins have been previously tested for HSV treatment. Three sesquiterpene coumarins badrakemin acetate (1), kellerin (2) and samarcandin diastereomer (3) were isolated from the gum resin of *Ferula assa-foetida*, a herbal medicine with antimicrobial, antiprotozoal and antiviral effects. Compounds were identified by 1D and 2D- NMR spectroscopies and comparison with literature data. A comparative evaluation of cytotoxicity and antiviral activity showed that kellerin (2) could significantly inhibit the cytopathic effects and reduce the viral titre of the herpes virus type 1 (HSV-1) DNA viral strain KOS at concentrations of 10, 5 and 2.5 µg/mL.

## Introduction

HSV-related opportunistic infections are involved in the development of various malignancies, and there is a growing need to find new antiviral compounds to address the emergency of drug-resistant viral strains and to improve the efficacy and tolerability of our current antiviral armoury (1-2), that, in the case of herpes virus, include both nucleoside analogues (acyclovir) and non-nucleoside HSV-inhibitors (3). Several studies have shown the potential of herbal medicine to afford new antiviral leads that can inhibit viral replication, viral genome synthesis, or both processes (4-5). Remarkably, several isoprenoids show antiviral activity against type 1 or 2 herpes virus (6-9), sometimes comparable to that of acyclovir, the golden standard of the field (10). The potent activity of many sesquiterpene coumarins toward several strains of influenza virus (11) and rhinoviruses (12) has prompted us to extend these studies to their anti-herpetic action, focusing on asafoetida as a source of these compounds.

Asafoetida is the gum resin obtained by incision of the collar of roots of several *Ferula *spp (Apiaceae) characterized by foul smell. Asafetida has been extensively investigated from a phytochemical standpoint, and sesquitepene coumarin ethers (13-14), sesquiterpenes (15-16) and sulphides (17-18) have emerged as its hallmark constituents. Sesquiterpene coumarins such as, assafoetidnol A, assafoetidnol B, gummosin, polyanthin, badrakemin, neveskone, samarcandin, galbanic acid (13), 5-hydroxyumbelliprenin, 8-hydroxyumbelliprenin and 8-acetoxyumbelliprenin (14) have been previously reported from the plant, mostly from samples far away from original place of the plant. Since asafoetida is a commercial umbrella name that lacks a specific botanical connection, it is difficult to assign the occurrence of the various compounds to a particular *Ferula* species. To overcome this limitation, we have started a systematic study of various foul-smelling gum resins obtained from botanical collections of Iranian *Ferula* species, trying to bridge the gap between the existence of multiple botanical sources for commercial asafetida and the occurrence of a specific phytochemical pattern and bioactivity profile in its various plant sources. Although complicated by the likely occurrence of chemotypes, we feel this approach is a prerequisite for a better exploitation of the biomedical profile of asafetida and the establishment of a botanically certified supply chain of the product. We present here the results obtained with the gum-resin of *F. assa-foetida*, focusing on its sesquiterpene coumarin profile and their antiviral activity. The exudates from this plant is locally known as “Anghuzeh”, “Heng” and “Buganeh”, and has traditionally been used for a bewildering range of ailments (epilepsy, urinary, gasterointestinal and respiratory infections), as well as an aphrodisiac (19-20), an emmenagogue (21), and to treat snake and insects bites (22), with the best documented folk use being the management of intestinal worm infections. The growing interest for the antiviral activity of natural products and effectiveness of sesquiterpene coumarins on some viral infections (11) provided a rationale for focusing on this end-point.

## Experimental

*General instruments*

High performance liquid chromatography (HPLC) was performed on a Waters^®^ apparatus equipped with a pump module 600 and a dual wavelength (254, 366 nm) UV detector using Shimpack^®^ Si 20 X 250 columns. The NMR spectra were recorded on a Brucker^®^ (400 MHz) instrument, using CDCl_3_ as solvent. Homonuclear ^1^H connectivities were determined by the COSY experiment. One-bond heteronuclear ^1^H-^13^C connectivities were determined with the HSQC experiment. Through-space ^1^H connectivities were established using ROESY experiments with a mixing time of 300 ms. Two- and three-bond ^1^H-^13^C connectivities were determined by gradient 2D HMBC experiments optimized for ^2,3^*J* = 7.7 Hz. MS analysis was obtained on an Agilent 6410 Triple Quadrupole mass spectrometer (Agilent Technologies, Palo Alto, CA, USA) coupled to an Agilent Mass Hunter Workstation B.01.03. Silica gel in different particle sizes was used for gravity column chromatography. TLC plates (Silica gel 60 GF_254_ precoated plates, Merck) were revealed by UV observation at 254 and 365 nm, and by spraying with cerium sulfate/ molybdate. All reagent solid material and solvents were purchased from Merck (Germany).

*Plant material*

The gum-resin of *Ferula assa-foetida *was collected by an incision of the plant root in Toghrol Jerd region, Kerman, Iran, in May 2011 at an altitude of 2200 m above sea level. The plant was identified by Dr. Mohammad-Reza Kanani, Department of Biology, Medicinal Plants and Drugs Research Institute, Shahid Beheshti University, Tehran, Iran, and compared to voucher specimen of the source plant (No. MPH-1251) deposited at aforementioned institute. 

*Extraction and isolation of compounds*

**Figure 1:**
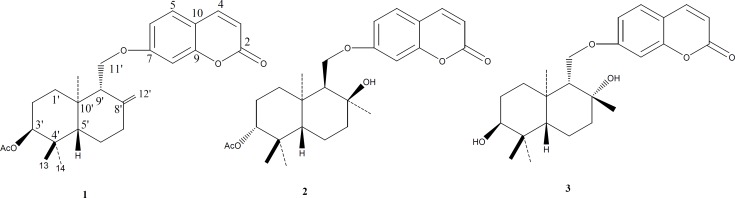
Structures of the sesquiterpene coumarins from *Ferula assa-foetida* oleo-gum-resin. 1**: **badrakemin acetate, 2: kellerin, 3**:** samarcandin diastereomer

The gum resin (140 g) was dried in dark, and then extracted with hexane (2 x 0.9 L, constant stirring for two days) to remove the non-polar constituents. The defatted material was then extracted with MeOH (2 x 1 L), in which after evaporation at reduced pressure yielded 79 g of a reddish resinous residue, part of which (10 g) was partitioned between aqueous methanol (40 mL water in 140 mL MeOH) and *n*-hexane (100 mL). The polar phase was evaporated and fractionated by gravity column chromatography on silica gel, using a heptane-EtOAc gradient. Further purification of the primary fractions was achieved by normal phase HPLC to get 1 (3 mg, heptane-EtOAc 1:1), 2 (48 mg, heptane-EtOAc 3:7) and 3 (8 mg) (Figure 1). 

Compound 2. ESI-MS m/z 442 [M^+^]. NMR data in Table 1.

**Table 1:** ^1^H (400 MHz) and ^13^C NMR (100 MHz) spectral data for compound 2 (CDCl_3_)**.**

**Atom No.**	**C**	**H (** ***J*** ** in Hz)**
Ac C=O	170.4, q^a^	-
7	161.8, q	-
2	161.2, q	-
9	155.9, q	-
4	143.4, s	7.65,d (9.2)
5	128.8, s	7.380,d (8.4)
3	113.28, s	6.250,d (9.2)
6	112.85, s	6.860,dd (8.4, 2.4)
10	112.58, q	-
8	101.11, s	6.820,d (2.4)
3	78.48, s	4.624, bs
10'	37.71, q	-
11	67.66, d	4.171, dd (10.1) 4.165, dd (10.1)
9	57.80, s	1.537, bs
5	43.59, s	1.934, m
1	39.69, d	1.720, m 1.756
8'	73.50, q	-
4	36.85, q	-
12	31.55, t	1.320, s
7	30.74, d	1.756, d (2.8)
13	28.20, t	0.892, s
15	24.12, t	1.360, s
2	22.72, d	1.620, 1.580, dd (16, 2.8)
14	21.63, t	0.929, s
Ac CH3	21.05, t	1.780, s
6	17.96, d	1.690, m 1.475, m

^a^ Multiplicity was determined by DEPT experiments.

*Antiviral evaluation*

*Cells and viruses and viral infections*

African green monkey kidney cells (Vero cell line CCL-81-ATCC) were grown in Eagle minimum essential medium (MEM) supplemented with 10% (v/v) Fetal Calf Serum (FCS) (Gibco), 100 U mL^-1^ penicillin (Gibco) and 100 mg mL^-1^ Streptomycin (Gibco), 2 mM l-Glutamine (Gibco) and 1mM sodium pyruvate (Gibco). A virus stock of herpes simplex virus type I, strain KOS (University of Isfahan/ Iran) was prepared,in which Vero cells infected at a low multiplicity of infection, incubated for 4 days and virus containing supernatant was harvested every day after infection until 4 days. 

*Evaluation of cytotoxicity* 

The different concentrations of the isolated compounds were prepared and tested in the antiviral experiments. The solutions were prepared by dissolving the extracts in DMSO at sub toxic concentration (maximum of 0.019%). To assess the effect of compounds **1**, **2** and **3** on uninfected Vero cells, dilutions ranging from 2.5, 5, 10 µg/mL in the maintenance medium, were added to Vero monolayers (using a 96-well microplate with 4.0×10^4^ cells per well). After 72 h of incubation at 37 C, cytotoxicity was determined by XTT proliferation assay kit (Roche, Germany) according to the instruction (23). All assays were carried out in triplicate.

*Antiviral activity*

Anti-HSV activity was investigated using plaque forming assay. Dilutions of the extracts at concentrations of 10 and 5 and 2.5 µg/mL were added on confluent 24 h old monolayer of Vero cells grown in microtitre tissue culture plates just before virus inoculation. The cell monolayer was infected with 25 PFU of HSV1 and incubated at 37 C for 2 hours. The infected cells were washed and overlaid with medium supplemented with 2.5 % methylcellulose and different concentrations of extract. 0.1% DMSO was used as negative control. After 3-4 days, the overlay medium was removed and the cell monolayer was stained at room temperature. Finally, cell monolayer was fixed with 3.7% formalin for 5 min and visible plaques were counted after staining with 1% crystal violet. The antiviral activity was determined by the following formula:

$\mathrm{Percentage of Inhibition}=\left[1-\frac{{\left(\mathrm{number of plaque}\right)}_{\mathrm{tested}}}{{\left(\mathrm{number of plaque}\right)}_{\mathrm{control}}}\right]\times 100$

The minimal concentration of extracts required to suppress the formation of virus plaque number by 50% (IC50) was calculated by regression analysis of the dose response curve generated from data (24). 

## Results and Discussion

A combination of open column chromatography and HPLC of the defatted MeOH extract of the oleo-gum-resin of *Ferula assa-foetida*, resulted in the isolation of three sesquiterpene coumarins, namely kellerin (1), badrakemin acetate (2) and samarcandin diastereomer (3) (Figure 1). The structures of known compounds were confirmed by direct comparison of their spectral data (^1^H NMR, ^13^C NMR and DEPT) with those reported in literature. As an instance, the structure elucidation of compound 2 is discussed here. 

Regarding ^1^H NMR spectrum of compound 2, proton signals of umbelliferone part appeared at δ_H_ 6.25 (H-3), 7.65 (H-4), 7.38 (H-5), 6.86 (H-6) and 6.82 ppm (H-8). Analysis of its ^13^C NMR spectrum (CDCl_3_, Table 1) revealed the presence of 25 carbons, nine related to coumarin moiety including lactone carbonyl δ_C_ 161.2 ppm (C-2), fifteen carbons related to sesquiterpene moiety and one for acetate ester at δ_C_ 170.5. DEPT 90 and 135° analysis showed five methyls, four aliphatic methylenes, eight methines including coumarin carbons at δ_C_ 113.3 (C-3), δ_C_ 143.4(C-4), δ_C_ 128.8 (C-5), δ_C_ 112.8 (C-6), δ_C_ 101.1 (C-8) and one primary alcoholic methylene at δ_C_ 67.6 (C-11'). There were no olefinic methylenes. 

The sesquiterpene moiety was determined from the primary alcohol protons at δ_H_ 4.17 (H-11'a) and 4.46 (H-11'b), the geminal proton to ester group at δ_H_ 4.62 (H-3', s) and four methyl groups at δ_H_ 1.36 (H-12', s), 0.89 (H-13', s), 0.93 (H-14', s) and 1.32 (H-15', s).

All the proton resonances were then associated to those of the relevant carbon atoms by 2D HSQC experiment. The ^1^H–^1^H COSY spectrum showed that the methine proton at δ_H_ 4.62 (H-3') is coupled to the methylene at δ_H_ 1.69 (H-2'). 

The inspection of ^2,3^*J*_H,C_ HMBC spectrum helped us to determine different moieties connectivity. In particular, the proton at δH 4.62 (H-3') showed long-range correlations with the carbon resonances at δC 170.5 (C-1"), which clearly places the acetate moiety at C-3'. Besides, there was a long range correlation between tertiary carbon at δC 57.8 (C-9') and two methyls at δH 1.32 (H-15') and 1.36 (H-12'). 

**Figure 2:**
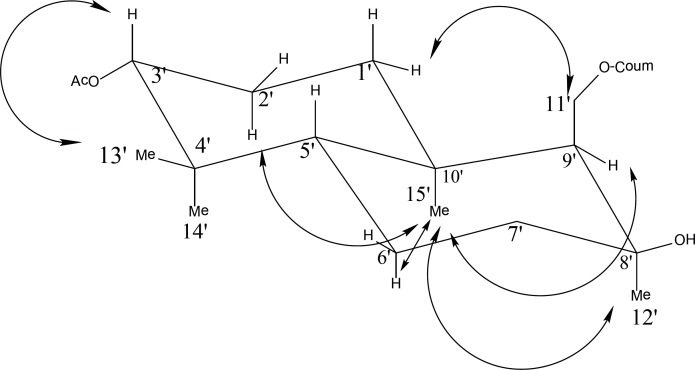
Selective NOE correlations of compound 2 (kellerin)**.**

The relative stereochemistry of the chiral centers could be established by examining the various cross peaks in the ROESY spectrum, Fig. 2. The ROESY experiment supported the proposed stereochemistry. H-2'_ax_ showed cross-peaks with H-14'_ax_, H-15'_ax_, and H-1'_eq_. Besides, H-5'_ax_ exhibited cross-peaks with H-13'_eq_, H-1'_ax_, and H-9'_eq_, but no correlation was seen between H-9'_eq _and H-5'_ax_, which would propose α equatorial position for H-9' (Figure 2). Furthermore, methyl group protons of the acetyl group appear at unusually high field, in which this diamagnetic shift could only be seen due to the screening effect of the coumarin ring when CH2-OAr group is axially oriented (25). 

**Figure 3:**
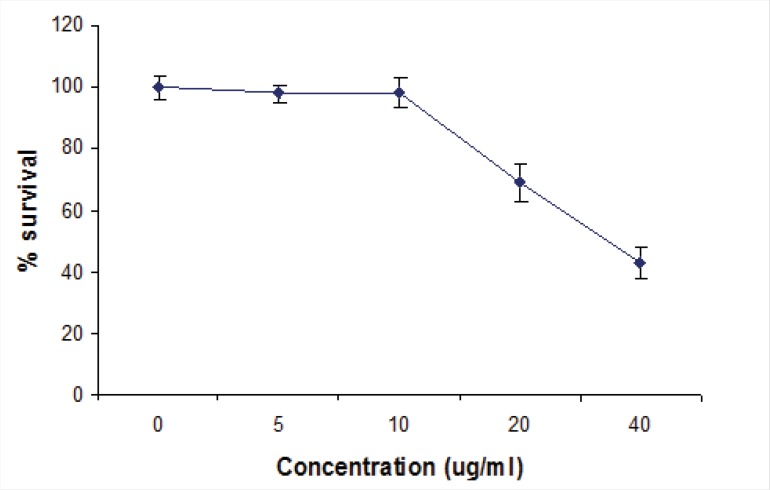
Cytotoxic activity of compound 2. Cytotoxicity on Vero cells were measured by XTT assay. Data are expressed as means ± standard deviations (P< 0.05).

Compound 2 could not affect the viability of Vero cells as evaluated by the XTT assay. Results showed that kellerin had no cytotoxic effect up to the concentration of 10 µg/mL (Figure 3). 

**Figure 4:**
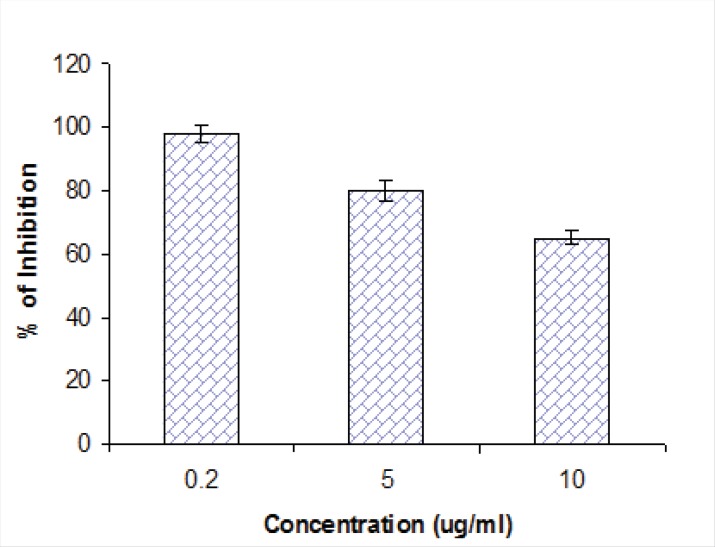
Effect of compound 2 on HSV-1 replication in Vero cells. The 50% inhibitory concentration (EC_50_) of each extract was calculated using regression line. Each bar represents the mean ± SD of three independent experiments.

The antiviral activity of all compounds isolated was evaluated by plaque reduction assay. Among the isolated compounds, only compound 2 could reduce the viral titre of the HSV-1 DNA viral strains KOS at concentrations of 10, 5 and 2.5 µg/mL (Table 2). This effect on HSV-1 replication was quantified through the reduction of the infectious titre after several rounds of multiplication. Results showed that at the concentration of 10, 5 and 2.5 µg/L, compound **2** inhibited HSV-1 multiplications with an inhibition rate of 98 ± 5.2%, 80% and 65%, respectively. Kellerin was effective in EC50 as 38 µg/mL (Figure 4).

(*P* < 0.05).

For comparison, the effect of the reference compound ACV was also studied under our assay conditions. When compared to the reference compound ACV, 2 was significantly less potent (Table 2) (26). 

**Table 2:** The percentage of antiviral activity of pure sesquiterpene coumarins determined as plaque reduction assay by comparison with untreated controls. The values are expressed as mean ± SD of three separate experiments

**Sample**	**10**	**5**	**2.5**
1	-	-	-
2	98 ± 5.2%	80 ± 3.8%	65 ± 2.2%
3	-	-	-
Acyclovir	100	100	85 ± 2.9%

Several natural products are effective against HSV-1 or -2 like several mono, di, tri (6-8) and tetra terpenoids (9), but no sesquiterpene coumarins have been previously investigated for HSV treatment, despite their effect on HIV (27). Different mechanisms underlie the anti-HSV activity of natural products. Essential oils directly inactivate herpes virus and might interfere with virion envelope structures or mask viral structures necessary for adsorption or entry into the host cells, while inhibition of HSV occur before adsorption but not after penetration of the virus into the cell. Inactivation could occur by prevention of cell-to-cell spread (6). Polyphenols like tannins (28) and flavonoid glycosides (29,30) exhibit anti-HSV activity mostly by inhibiting entry into the host cells. Other mechanisms are diminishing the synthesis of viral DNA and inhibition the spread of infectious viral particles by using HSV-1 expressing -galactosidase activity as a detection system (8) or oligomerization of HSV-1 glycoprotein D (28). Oligomeric stilbenoids exert anti-HSV effect by ROS production promotion (31). Most sesquiterpenoids are only moderately virucidal against different enveloped viruses, *e.g*. herpes simplex, cytomegalo-, measles and influenza viruses, but several essential oils show virucidal activity through inhibition of glycosylation of viral proteins (6). A remarkable aspect of the antiviral activity of natural products is their ability to inhibit acyclovir-resistant HSV-1 isolates, showing mechanism complementarity with acyclovir that interferes with the DNA polymerase inside the cell (6,32). Furthermore, some plant extracts show only indirect antiviral properties, being endowed of immunomodulatory properties by interacting with IL-12, IFN- and TNF-α (9). Overall, should be considered that *Ferula spp *render various biologically active components (33-35).

Regarding the mechanism of action of sesquiterpene coumarins, given the capacity of coumarin to trap thiol groups and act as a Michael acceptor (36), it does not seem unreasonable to assume that they share the same mechanism as sesquiterpene lactones (10). 
